# Copper complexes as candidate therapeutic agents against infiltrative glioblastoma cells

**DOI:** 10.1039/d6md00801a

**Published:** 2026-10-15

**Authors:** Thomas Weston, Kayley Mulhall, Sophie Melling, Dylan Smith, Stephen P. Argent, Konstantinos-Panagiotis Karadimas, Cara Moloney, Une Kontrimaite, Harry Porter, Ruman Rahman, Luisa Ciano, Phoebe McCrorie

**Affiliations:** a School of Chemistry, University of Nottingham University Park NG7 2RD UK luisa.ciano@nottingham.ac.uk; b Biodiscovery Institute, School of Medicine, University of Nottingham University Park NG7 2RD UK Phoebe.McCrorie1@nottingham.ac.uk; c School of Pharmacy, University of Nottingham University Park NG7 2RD UK

## Abstract

Copper-based compounds have long been explored for their anti-cancer potential and other biological effects. While positive results have been reported, their use as cytotoxic agents remains limited; additionally, novel ligands and complexes could provide alternative strategies for cancer treatment. In this work, we synthesised three Schiff base ligands and their corresponding copper(ii) complexes; the ligands included a non-substituted, an electron-withdrawing or an electron-donating substituent positioned *para* to the Cu-coordinating hydroxyl group to evaluate how these modifications might influence cytotoxicity. The complexes were assessed against highly invasive glioblastoma (GBM) cells grown in both 2D monolayers and 3D spheroid models. GBM is a grade 4 brain tumour with an extremely poor prognosis, and patient survival has shown little improvement over the past two decades, underscoring the urgent need for new therapeutic strategies. Our synthesised copper(ii) complexes demonstrated strong cytotoxic activity across four patient-derived GBM cell lines isolated from the tumours' invasive margin. All three compounds exhibited low micromolar potency, with IC_50_ values below 10 μM in 2D cultures and below 20 μM in 3D spheroids. Treatment with the complexes led to a marked increase in reactive oxygen species, suggesting oxidative stress as a key component of their mechanism. However, caspase-3/7 activation remained minimal, indicating that cell metabolic impairment was not significantly associated with caspase-3/7-mediated apoptosis. This study demonstrates that Schiff base copper(ii) complexes hold meaningful therapeutic potential against a disease for which current outcomes remain devastating.

## Abstract 2

Copper complexes yielded low μM IC_50_ values in invasive glioblastoma cell lines. Created in BioRender. McCrorie, P. (2026) https://BioRender.com/gs8z4yq.

## Graphical abstract

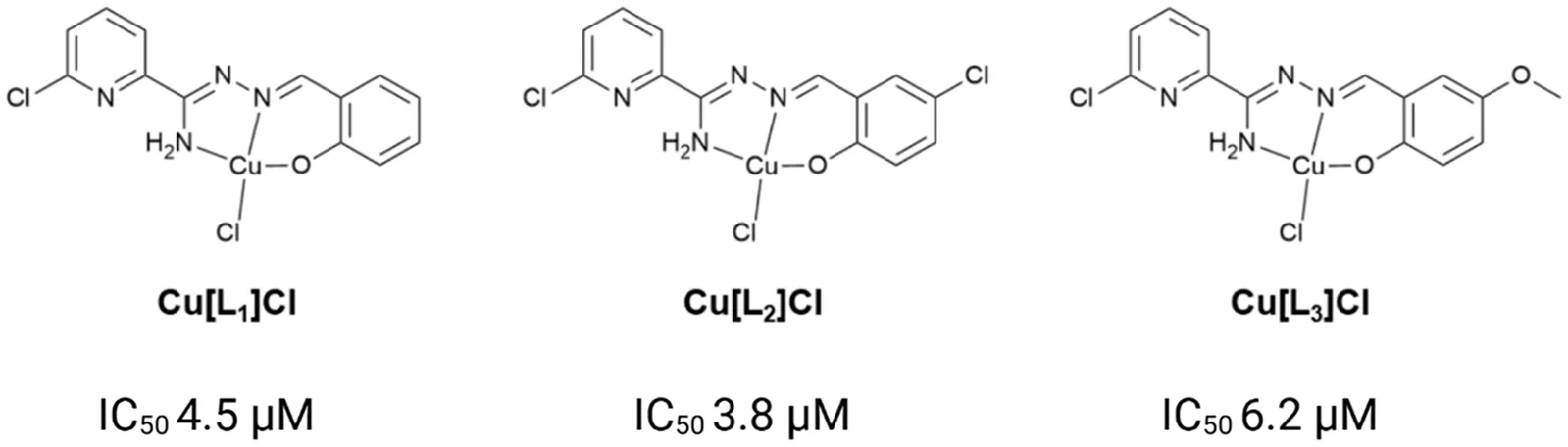

## Introduction

Glioblastoma (GBM) is a grade 4 brain tumour with a median survival of approximately 12–15 months, despite a multimodal treatment approach that includes maximal safe surgical resection followed by concurrent chemoradiotherapy.^1^ Standard care often involves oral temozolomide, though its therapeutic benefit is largely influenced by the tumour's O-6-methylguanine-DNA methyltransferase (MGMT) promoter methylation status.^2^ Despite widespread adoption of this regimen, overall survival outcomes have remained stagnant since 2005, underscoring the urgent need for novel interventions.

Metal complexes such as cisplatin and carboplatin have long been used as chemotherapeutic agents. However, their clinical use is limited by significant toxicity, including gastrointestinal disturbances, nephrotoxicity, and hepatotoxicity.^3^ Therefore, research into less toxic metal complexes is critical, especially those utilising endogenous metal ions. Cancer cells require large amounts of copper to assist their tumour progression *via* angiogenesis, growth and metastasis.^4^ As a result, tumour cells try to enrich their copper content, which leads to copper overload and cell death in the presence of excess copper and/or copper complexes.^5,6^ These complexes can induce cancer cell death *via* many studied mechanisms, including oxidative stress from the generation of reactive oxygen species (ROS), inhibition of proteasomes and topoisomerases I/II, or by inducing cuproptosis – a specialised form of programmed cell death triggered by excess copper.^7,8^

Hence, copper(ii) complexes are of interest in the development of novel cancer therapies due to their varying pharmacological effects.^9,10^ In particular, Schiff base-derived copper(ii) complexes have gained considerable interest as candidates for novel cancer therapies owing to their straightforward synthesis, structural versatility, and tuneable redox behaviour.^11^ Pyridine-, pyrazine-, and salicylaldehyde-based ligands have become increasingly prominent due to their favourable coordination characteristics and broad spectrum of biological activities.^12–14^ Numerous studies have reported the promising anti-cancer potential of tridentate Schiff base copper(ii) complexes incorporating pyridine and phenolic groups.^15–19^ Additionally, the substantial influence of ligand substituents on biological activity has been well documented, underscoring the highly tuneable nature of these compounds.^20,21^

Previous studies have demonstrated efficacy of copper-based therapeutics and complexes against GBM cell lines derived from the proliferative tumour core;^22^ Morelli *et al.* conjugated the bifunctional species bis(3,5-dimethylpyrazol-1-yl)-acetate and the drug amantadine, which was used as a chelator for the synthesis of new copper complexes. The study found that their copper complexes generated IC_50_ values significantly lower than cisplatin. Furthermore, anti-cancer effects were prevented by treatment with the copper chelator tetrathiomolybdate, indicating copper mediated cell death. The study concluded that the complexes were able to increase ROS production, leading to DNA damage and cell death.^23^ Additionally, Anjomshoa *et al.* developed an aqueous nanosuspension of a Cu(ii) complex, [Cu(^2MeO^bpy)_2_](PF_6_)_2_, which had enhanced water solubility properties. Cytotoxicity was comparable to cisplatin, and the complex conferred anti-migratory activity in U-87 GBM cells.^24^ Although these studies highlight the potency of copper complexes against GBM core cell lines, such cells originate from tissue typically removed during surgical resection. Emerging evidence suggests that invasive margin cells more accurately reflect the recurrent tumour responsible for patient mortality.^25^

In this context, this study seeks to investigate the anti-cancer activity of three Schiff base copper(ii) complexes against clinically-accurate patient-derived GBM invasive margin cells, utilising both 2D and 3D culture models. We present the synthesis and characterisation of three novel *N*,*N*,*O*-tridentate Schiff base ligands (Fig. 1) and their corresponding copper(ii) coordination complexes. Given their facile synthesis, we aimed to investigate the tuneable properties of the complexes by including electron withdrawing or donating groups *para* to the Cu-coordinating hydroxyl group of the salicylaldehyde moiety to determine the putative anticancer properties of the complexes. The complexes and their corresponding metal-free ligands were tested on invasive glioblastoma cells in both 2D and 3D cultures, and their cytotoxic activity was evaluated alongside a preliminary investigation of their mechanism of action.

**Fig. 1:**
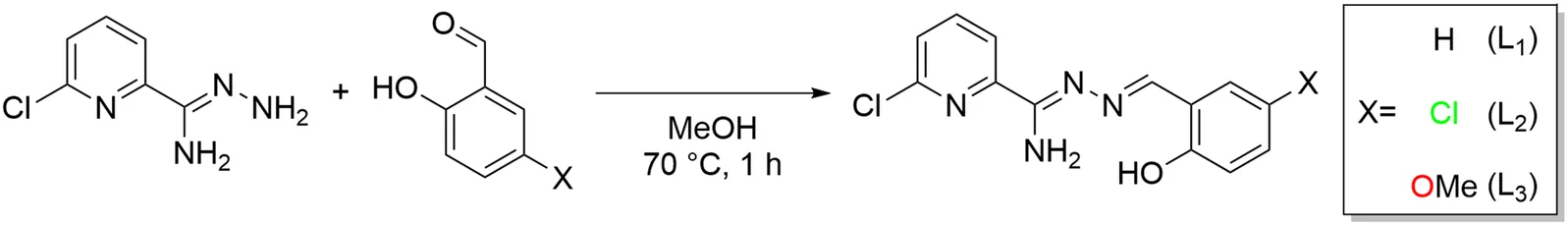
Reaction scheme for the synthesis of ligands L_1_–L_3_.

## Methods

### Ligands and copper complexes

All solvents and reagents were purchased from Merck, Fisher Scientific or Fluorochem. ^1^H and ^13^C NMR spectra were recorded at ambient temperature using a Bruker DPX400 (400.1 MHz for ^1^H and 100 MHz for ^13^C). Chemical shifts (*δ*) are reported in parts per million (ppm). High resolution mass spectra (HRMS) were recorded using a Bruker Compass MicroTOF Focus II mass spectrometer, using electro-spray ionisation (ESI) operating in positive ionisation mode.

#### Synthesis of 6-chloropicolinohydrazonamide

6-Chloropyridine-2-carbonitrile (250 mg, 1.80 mmol) was added to ethanol (4 mL), followed by addition of hydrazine hydrate (1.987 mL, 39.70 mmol); the solution was heated at reflux for 2 h. The resulting precipitate was filtered under vacuum yielding 6-chloropicolinohydrazonamide as a white solid (290.4 mg, 94.9% yield): mp 208-210 °C. ESI-MS: *m*/*z* calculated for [C_6_H_7_N_4_Cl^+^] 171.6000, found 171.0440. ^1^H-NMR (DMSO-*d*_6_) *δ* 7.88 (dd, 1H); 7.79 (t, 1H); 7.42 (dd, 1H); 5.64 (s, 2H); 5.51 (s, 2H). ^13^C-NMR (DMSO-*d*_6_): *δ* 153.3 (C); 149.1 (C); 142.1 (C); 140.1 (CH); 123.4 (CH); 118.5 (CH). Elemental analysis (%): for C_6_H_7_N_4_Cl calculated: C 42.23, H 4.14, N 32.84; found: C 42.16, H 4.01, N 32.98.

#### Synthesis of L_1_ (6-chloro-*N*′-((*E*)-2-hydroxybenzylidene)picolinohydrazonamide)

6-Chloropicolinohydrazonamide (100 mg, 0.588 mmol) was added to methanol (2 mL). Salicylaldehyde (92.07 μL, 0.882 mmol) was added and the reaction heated at reflux for 1 h. The reaction was cooled on ice and the resulting precipitate was filtered under vacuum yielding the final product as a yellow solid (113.7 mg, 70.4% yield). Melting point: 148–150 °C. ESI-MS: *m*/*z* calculated for [C_13_H_11_N_4_OCl^+^] 275.0694, found 275.0699. ^1^H NMR (CDCl_3_): *δ* 11.60 (s, 1H); 8.65 (s, 1H); 8.28 (dd, 1H); 7.78 (t, 1H); 7.45 (dd, 1H); 7.36 (td, 2H); 7.04 (m, 1H); 6.97 (td, 1H); 6.10 (s, 2H). ^13^C-NMR (CDCl_3_): *δ* 160.1 (CH); 159.2 (C); 153.7 (C); 150.6 (C); 150.4 (C); 139.3 (CH); 132.0 (CH); 131.7 (CH); 125.9 (CH); 120.0 (CH); 119.6 (CH); 118.5 (CH); 116.7 (CH). Elemental analysis (%) calculated: C 56.84, H 4.04, N 20.40; found: C 56.76, H 3.95, N 20.22.

#### Synthesis of L_2_ (6-chloro-*N*′-((*E*)-5-chloro-2-hydroxybenzylidene) picolinohydrazonamide)

6-Chloropicolinohydrazonamide (100 mg, 0.588 mmol) was added to methanol (2 mL). 5-Chlorosalicylaldehyde (138.1 mg, 0.882 mmol) was added and the reaction was heated at reflux for 1 h. The precipitate was filtered under reduced pressure yielding a yellow solid (164.1 mg, 90.6% yield). Melting point: 228–230 °C. ESI-MS: *m*/*z* calculated for [C_13_H_10_N_4_OCl_2_^+^] 309.0304, found 309.0303. ^1^H-NMR (DMSO-*d*_6_): *δ* 10.74 (s, 1H); 8.67 (s, 1H); 8.21 (dd, 1H); 8.00 (t, 1H); 7.94 (d, 1H); 7.68 (dd, 1H); 7.30 (dd, 1H); 7.15 (s, 2H); 6.95 (d, 1H). ^13^C-NMR (DMSO-*d*_6_): *δ* 156.6 (CH); 155.3 (C); 153.5 (C); 151.7 (C); 149.8 (C); 141.1 (CH); 131.3 (CH); 128.7 (CH); 126.4 (CH); 123.5 (C); 122.3 (CH); 120.9 (C); 118.4 (CH). Elemental analysis (%) calculated: C 50.51, H 3.26, N 18.12; found: C 50.68, H 3.04, N 17.73.

#### Synthesis of L_3_ (6-chloro-*N*′-((*E*)-2-hydroxy-5-methoxybenzylidene) picolinohydrazonamide)

6-Chloropicolinohydrazonamide (100 mg, 0.588 mmol) was added to methanol (2 mL). 2-Hydroxy-5-methoxybenzaldehyde (110.92 μL, 0.882 mmol) was added and the reaction was heated at reflux for 1 h. The precipitate was filtered under vacuum yielding the product as a yellow solid (136.4 mg, 76.1% yield). Melting point: 178–180 °C. ESI-MS: *m*/*z* calculated for [C_14_H_12_N_4_O_2_Cl^+^] 305.0800, found 305.0805. ^1^H-NMR (DMSO-*d*_6_): *δ* 8.70 (s, 1H); 8.22 (dd, 1H); 7.99 (t, 1H); 7.66 (dd, 1H); 7.34 (d, 1H); 7.06 (s, 2H); 6.90 (m, 2H); 3.75 (s, 3H). ^13^C-NMR (DMSO-*d*_6_): *δ* 155.9 (CH); 154.7 (C); 152.6; 152.2 (C); 151.8 (C); 149.8 (C); 141.0 (CH); 126.3 (CH); 120.9 (CH); 120.4 (C); 119.2 (CH); 117.5 (CH); 113.6 (CH); 56.0 (CH_3_). Elemental analysis (%) calculated: C 55.18, H 4.30, N 18.39; found: C 55.15, H 4.27, N 18.23.

#### Synthesis of copper complexes

The Cu(ii) complexes **Cu[L**_**1–3**_**]Cl** were prepared by mixing 1.0 mM L_1–3_ with 1.0 mM CuCl_2_ in 1 mL of DMSO. A colour change from yellow to green was observed upon addition of the copper solution to the ligand. The solutions were used for the activity tests without further isolation of the complexes. ESI-MS (Bruker microTOF II) *m*/*z* calculated for [**Cu[L**_**2**_**]**^**+**^] (C_13_H_9_Cl_2_CuN_4_O^+^) 369.9444, found 369.9445; calculated for [**Cu[L**_**3**_**]**^**+**^] (C_14_H_12_ClCuN_4_O_2_^+^) 365.9939, found 365.9941. It was not possible to obtain an ESI-MS spectrum for **Cu[L**_**1**_**]Cl**, complex formation is supported by X-ray crystallography, UV–vis and EPR data. CW-EPR (X-band, 77 K): **Cu[L**_**1**_**]Cl***g*_*z*_ = 2.248, |*A*_*z*_| = 534 MHz; **Cu[L**_**2**_**]Cl***g*_*z*_ = 2.242, |*A*_*z*_| = 535 MHz; **Cu[L**_**3**_**]Cl***g*_*z*_ = 2.236, |*A*_*z*_| = 546 MHz.

### Single crystal X-ray diffraction

Single crystals suitable for X-ray diffraction were obtained by the slow evaporation of concentrated solutions of ligands **L**_**1**_**–L**_**3**_ in methanol at room temperature. For the Cu-complexes, single crystals were obtained by the addition of CuCl_2_ in a 1 : 1 concentration ratio to a solution of ligand in methanol, and slow evaporation of the solvent at room temperature yielded crystals suitable for X-ray diffraction. Combined CIF reports for the structures obtained can be found in Table S1.

### Single crystal X-ray diffraction experimental procedure

Single crystals were selected and mounted using Fomblin® (YR-1800 perfluoropolyether oil) on a polymer-tipped MiTeGen MicroMountTM and cooled rapidly to 120 K in a stream of cold N_2_ using an Oxford Cryosystems open flow cryostat.^26^ Single crystal X-ray diffraction data for **Cu[L**_**1**_**]Cl-MeOH** and **L**_**2**_**-H**_**2**_**O** were collected on an Oxford Diffraction GV1000 (TitanS2 CCD area detector, mirror-monochromated Cu-Kα radiation source; *λ* = 1.54184 Å, *ω* scans). Single crystal X-ray diffraction data for **Cu[L**_**3**_**]Cl-H**_**2**_**O** and **L**_**3**_**-H**_**2**_**O** were collected on an XtaLAB PRO MM007 (PILATUS3 R 200 K Hybrid Pixel Array detector, mirror-monochromated Cu-Kα radiation source; *λ* = 1.54184 Å, *ω* scans). Cell parameters were refined from the observed positions of all strong reflections and absorption corrections were applied using a Gaussian numerical method with beam profile correction (CrysAlisPro).^27^ Structures were solved within Olex2 (ref. 28) by dual space iterative methods (SHELXT)^29^ and all non-hydrogen atoms refined by full-matrix least-squares on all unique *F*^2^ values with anisotropic displacement parameters (SHELXL).^30^ Structures were checked with checkCIF *via*https://checkcif.iucr.org/ (Table S1). CCDC-2421573–2421576 and 2571886 contain the SI for these compounds.

### Single crystal X-ray diffraction refinement details

All hydrogen atoms were observed in the electron density maps of the structures. Hydrogen atoms bound to oxygen and nitrogen atoms were refined with their O–H and N–H distances restrained to target values of 0.84 and 0.88 Å, respectively (DFIX, esd 0.02 Å). All other hydrogen atoms were geometrically placed and refined with a riding model.

### Job's plot (method of continuous variation)

The stoichiometry of the copper–ligand complexes was determined by a Job's method (Method of Continuous Variation). Solutions of L_1–3_ and CuCl_2_ were prepared at a concentration of 1.0 mM in DMSO. Solutions were mixed in varying molar ratios while maintaining a total concentration of 50 μM. Solutions were made up to 1.0 mL with DMSO and analysed by UV–vis spectroscopy on a Biotek Epoch 2 microplate reader monitoring at 430, 440 and 450 nm for complexes **Cu[L**_**1**_**]Cl**, **Cu[L**_**2**_**]Cl** and **Cu[L**_**3**_**]Cl**, respectively.

### EPR spectroscopy

Continuous wave (CW) X-band EPR spectra were recorded on a Bruker micro EMX spectrometer operating at ∼9.30 GHz with a modulation amplitude of 4 G, modulation frequency of 100 kHz and microwave power of 10.02 mW at 165 K; 8 scans were acquired for each sample. Spectral simulations were performed using EasySpin 6.0.6 (ref. 31) integrated into MATLAB R2024a. Samples (1.2 mM **L**_**1–3**_ and 1.0 mM **CuCl**_**2**_) were prepared in DMSO and flash frozen in liquid N_2_ prior to analysis. For the simulation, it was assumed that the *g* matrix and A tensors were axially coincident. While the exact values for the perpendicular region could not be determined solely from X band spectra, good simulations were obtained using the parameters outlined in Table S2. Simulations were improved by the inclusion of two nitrogen and one chloride nuclei, although the exact parameters could not be determined due to the lack of resolved superhyperfine coupling.

### Cell culture

Culture conditions were maintained at 37 °C with 5% CO_2_ and 90% relative humidity. GIN8 (**G**lioma **IN**vasive margin cells) were isolated from medial front invasive margin (54 y female, wild-type IDH (primary GBM), intact ATRX, 0% MGMT promoter methylation, 90% resection plus Gliadel wafers; treatment 60 Gy radiotherapy, concurrent and adjuvant temozolomide). GIN28 were isolated from the 5-aminolevulinic acid (5-ALA) fluorescence-positive invasive margin, (71 y male, wild-type IDH (primary GBM), intact ATRX, 0% MGMT promoter methylation 99% resection; no adjuvant therapy (patient choice)). GIN31 were isolated from the 5-ALA fluorescence-positive invasive margin (wild-type IDH (primary GBM), intact ATRX, 0% MGMT promoter methylation, 100% resection; treatment 60 Gy radiotherapy, concurrent and adjuvant temozolomide). GIN38 were isolated from the 5-ALA fluorescence-positive invasive margin (69 y male; wild-type IDH (primary GBM); intact ATRX; 0% MGMT promoter methylation; 100% resection; treatment 60 Gy radiotherapy, concurrent and adjuvant temozolomide). All cell lines were derived from tumours pre-treatment. Cell lines were cultured in Dulbecco's modified eagle medium (DMEM; Sigma-Aldrich, St. Louis, MO, USA) supplemented with 10% FBS, 1 g L^−1^ glucose and 2 mM l-glutamine (Sigma-Aldrich). Cells were used within a passage window of 5.

Human astrocytes (HA, ScienCell, #1800) were cultured as per supplier's protocol. Briefly, astrocytes were grown on poly-l lysine coated cultureware in astrocyte medium containing 2% FBS and 1% astrocyte growth supplement.

### 2D monolayer culture

To determine the IC_50_ for each copper complex, GIN cells were seeded into a tissue-culture treated 96-well plate at 5000 cells per well and incubated overnight at 37 °C. Copper complexes were synthesised as above, and added to cells at concentrations ranging from 0.5–10 μM. Cu alone and non-complexed ligands were tested at equivalent concentrations to delineate complex-derived efficacy. Cells were left for 72 hours at 37 °C. 5000 cells per well provided 90% confluency in the control well on the final day of the assay, whereby no cell aggregation was noted.

### 3D spheroid culture

Cells were seeded into a 96-well ultra-low attachment plate (Corning® Costar®, CLS7007) at 5000 cells per well and centrifuged at 300 × *g* for 5 minutes. Cells were then left overnight in the incubator to form spheroids. Copper complexes were then added to the spheroids at multiple concentrations between 1 μM and 50 μM, before being cultured for 72 hours at 37 °C with 5% CO_2_ and 90% relative humidity. At 72 h, control spheroids measure approximately 300 μm.

### Cell viability assays

Cells grown in 2D conditions (IC_50_ and biocompatibility tests) were analysed using a PrestoBlue™ assay as per product guidelines. Briefly, following 72 hours of exposure to copper complexes or ligand/copper-only, cell media was removed and 1x PrestoBlue™ in PBS was added to cells and incubated for 30 minutes at 37 °C. Fluorescence was measured on a TECAN Infinite M Plex plate reader at *λ*_Ex_ 544 nm and *λ*_Em_ 590 nm.

Cells grown as 3D spheroids were analysed using a CellTiter-Glo® 3D cell viability assay as per manufacturers guidelines. Briefly, after 72 hours of exposure to copper complexes, 100 μL media was slowly removed from the well and 50 μL CellTiter-Glo® 3D cell viability assay solution was added to each well. The covered plate was then shaken at 500 rpm for 5 minutes and incubated at room temperature in the dark for 25 minutes. 100 μL was transferred from each well into a white 96 well plate and the luminescent signal was read on a TECAN Infinite M Plex plate reader.

### ROS assay

Reactive oxygen species were measured in cells treated with IC_50_ and IC_75_ concentrations of each copper complex. GIN31 cells were seeded in a black, clear bottom 96-well plate at 5000 cells per well and left overnight to adhere, before drug stock was added to each well to achieve IC_50_ and IC_75_ concentration and left for 4 hours in the incubator. The ROS assay, CM-H_2_DCFDA (general oxidative stress indicator), was performed as per manufacturer's protocol, before being imaged on the EVOS M7000 microscope at 4× magnification. Images were analysed using ImageJ (fiji) and the fluorescence measured. The corrected total cell fluorescence was calculated using eqn (1) below. Three images were taken from 3 different wells and results calculated as mean ± SEM.

1 CTCF = Integrated density − (Area of selected cell × Mean fluorescence of background readings)

### Caspase 3/7 activity

Caspase 3/7 activity was monitored at 24 hours after the addition of IC_50_ concentrations of each drug using CellEvent™ Caspase-3/7 Green ReadyProbes™ Reagent and NucBlue™ Live ReadyProbes™ Reagent (Hoechst 33342) as per manufacturer's protocols. Each well was imaged on a NIKON Eclipse Ts2-Fl widefield microscope using the DAPI and GFP filter sets. Images were analysed using ImageJ (fiji) and the fluorescence measured. The corrected total cell fluorescence was calculated using eqn (1) above. Three images were taken per well and results calculated as mean ± SD.

### Statistical analysis

All data is displayed as mean ± SEM, *n* = 3, where * represents *p* < 0.05, ** represents *p* < 0.01, *** represents *p* < 0.001 and **** represents *p* < 0.0001. Significance was found using a one-way ANOVA with Dunnett's *post hoc* test.

## Results & discussion

### Synthesis and characterisation of ligands and Cu-complexes

The ligand scaffolds L_1–3_ were synthesised by Schiff base condensation of *para*-substituted salicylaldehyde derivatives with 6-chloropicolinohydrazonamide in methanol at 70 °C for 1 hour (Fig. 1). In all cases, the synthesis proceeded with high yields (>70%) and purity, and the ligands L_1–3_ were characterised by HRMS and NMR, while CHN analysis provided evidence of high purity (see Methods). Single crystals suitable for X-ray crystallography were obtained for **L**_**2**_**·H**_**2**_**O**, **L**_**2**_ and **L**_**3**_, showing an overall planar structure with two nitrogen and one oxygen atoms well positioned to coordinate a metal ion (Table S1 and Fig. S1). The copper(ii) complexes of ligands L_1–3_ were synthesised by the addition of a stoichiometric amount of CuCl_2_ to a solution of L_1–3_ in DMSO (Fig. 2). Addition of the copper(ii) salt to the ligands prompted a colour change from yellow to green, and the resulting complexes were analysed by UV–vis spectroscopy, EPR spectroscopy and, when possible, X-ray crystallography.

**Fig. 2:**
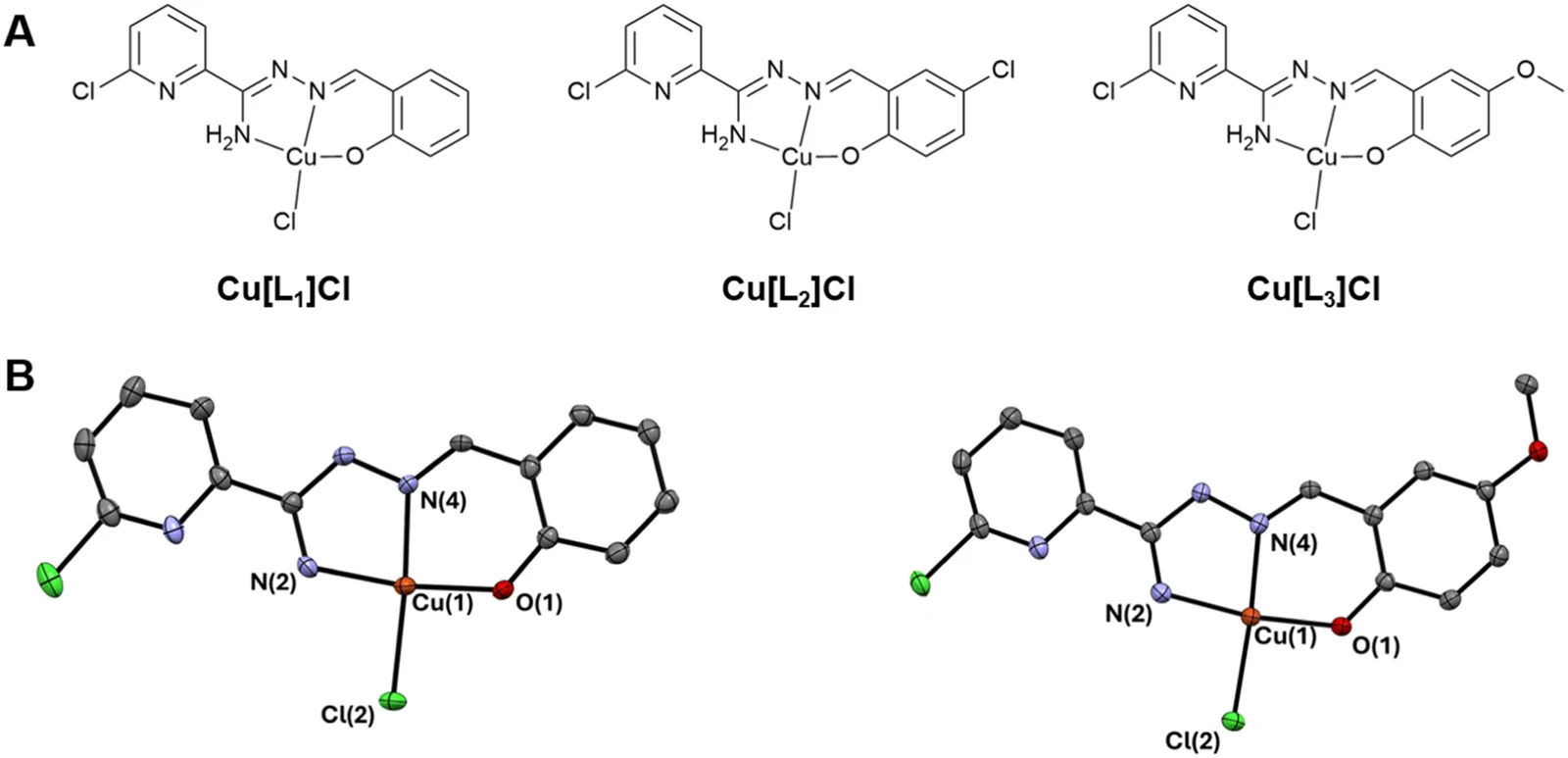
A) Structure of the Cu complexes. B) Ortep diagrams of **Cu[L**_**1**_**]Cl** (left) and **Cu[L**_**3**_**]Cl** (right). Hydrogen atoms and co-crystallised solvent residues have been omitted for clarity; carbon atoms are grey, oxygen red, chlorine green, nitrogen blue and copper orange.

Single crystals suitable for X-ray diffraction of the coordination compounds **Cu[L**_**1**_**]Cl** and **Cu[L**_**3**_**]Cl** were obtained by slow evaporation of concentrated solutions of the complexes in methanol. **Cu[L**_**1**_**]Cl** crystallises in the monoclinic *P*2_1_/*c* space group while **Cu[L**_**3**_**]Cl** crystallises in the orthorhombic *Pbca* space group. In both cases the metal centre shows a square planar geometry, with the copper coordinated by one oxygen and two nitrogen atoms from the ligand, while a chloride ion occupies the last coordination position (Fig. 2); both complexes have comparable bond lengths and angles around the copper centre, showing that the tridentate ligands provide the metal ion with a similar scaffold (Table 1). The geometry index (*τ*_4_)^32^ of the structures was calculated to be 0.11 and 0.13 for **Cu[L**_**1**_**]Cl** and **Cu[L**_**3**_**]Cl**, respectively, indicating a slight distortion of the square planar geometry.

**Table 1:** Key bond distance (Å) and angles (°) for **Cu[L**_**1**_**]Cl** and **Cu[L**_**3**_**]Cl**. The values in brackets next to each angle/distance represent the standard uncertainties

Bond angles (°)			Bond distances (Å)		
	**Cu[L**_**1**_ **]Cl**	**Cu[L**_**3**_ **]Cl**		**Cu[L**_**1**_ **]Cl**	**Cu[L**_**3**_ **]Cl**
Cl(2)–Cu(1)–O(1)	93.58(12)	90.53(4)	Cu(1)–Cl(2)	2.2201(14)	2.2570(5)
N(4)–Cu(1)–O(1)	92.02(16)	92.45(6)	Cu(1)–N(2)	1.921(4)	1.9213(16)
N(2)–Cu(1)–N(4)	80.69(17)	80.88(6)	Cu(1)–N(4)	1.962(4)	1.9546(15)
N(2)–Cu(1)–Cl(2)	93.84(14)	96.58(5)	Cu(1)–O(1)	1.896(3)	1.8928(13)
N(2)–Cu(1)–O(1)	172.56(18)	172.70(6)			
Cl(2)–Cu(1)–N(4)	173.63(12)	169.04(5)			

To ascertain that the crystal structures are representative of complexation behaviour in solution, the stoichiometry of the complexes was determined using a Job's plot, plotting absorbance at varying ratios of ligand : copper. All three complexes follow the same trend and show an absorbance maximum at *X*_a_ = 0.5, indicating a 1 : 1 stoichiometry (Fig. 3). These results not only confirm the crystallographic data, but also indicate that **Cu[L**_**2**_**]Cl**, for which single crystals could not be obtained, behaves in an equivalent manner to the other two complexes.

**Fig. 3:**
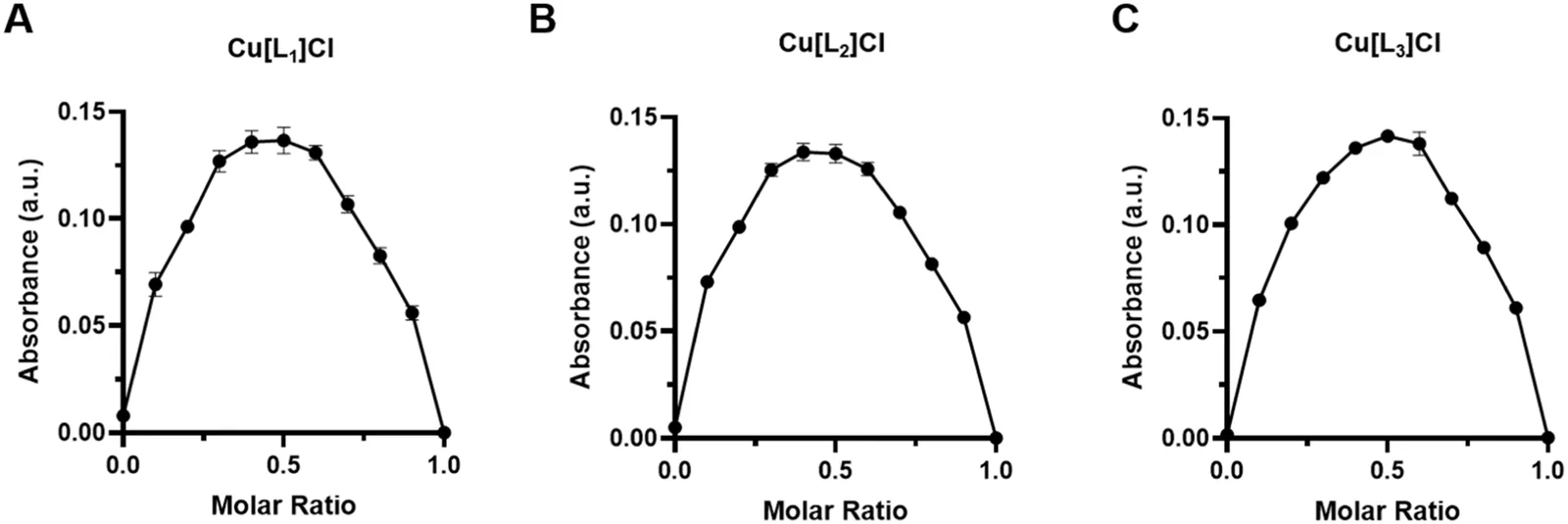
Job's analysis of A) **Cu[L**_**1**_**]Cl**, B) **Cu[L**_**2**_**]Cl**, and C) **Cu[L**_**3**_**]Cl**. Each experiment was performed in triplicate and the mean value is plotted.

Further investigation of the complexes **Cu[L**_**1–3**_**]Cl** was performed by continuous wave (CW) X-band EPR spectroscopy. CuCl_2_ was added to a solution of ligand in DMSO to reproduce the synthetic procedure, and the spectra were collected at 77 K. Fig. 4A–C shows the experimental and simulated EPR data. In all three cases, a single copper species is observed, with spin-Hamiltonian parameters consistent with square planar or Jahn–Teller elongated octahedral geometry (Table S2). It has to be noted that in all cases the simulations were improved by the inclusion of two coordinating nitrogen nuclei and a chlorine nucleus, but the absence of resolved superhyperfine coupling prevents the accurate determination of the number of coupled nuclei. The EPR data confirm the results of Job's plot and crystallography experiments; taken together, the data show that the ligands form a single copper species in a 1 : 1 ligand : metal ratio upon addition of the metal ion.

**Fig. 4:**
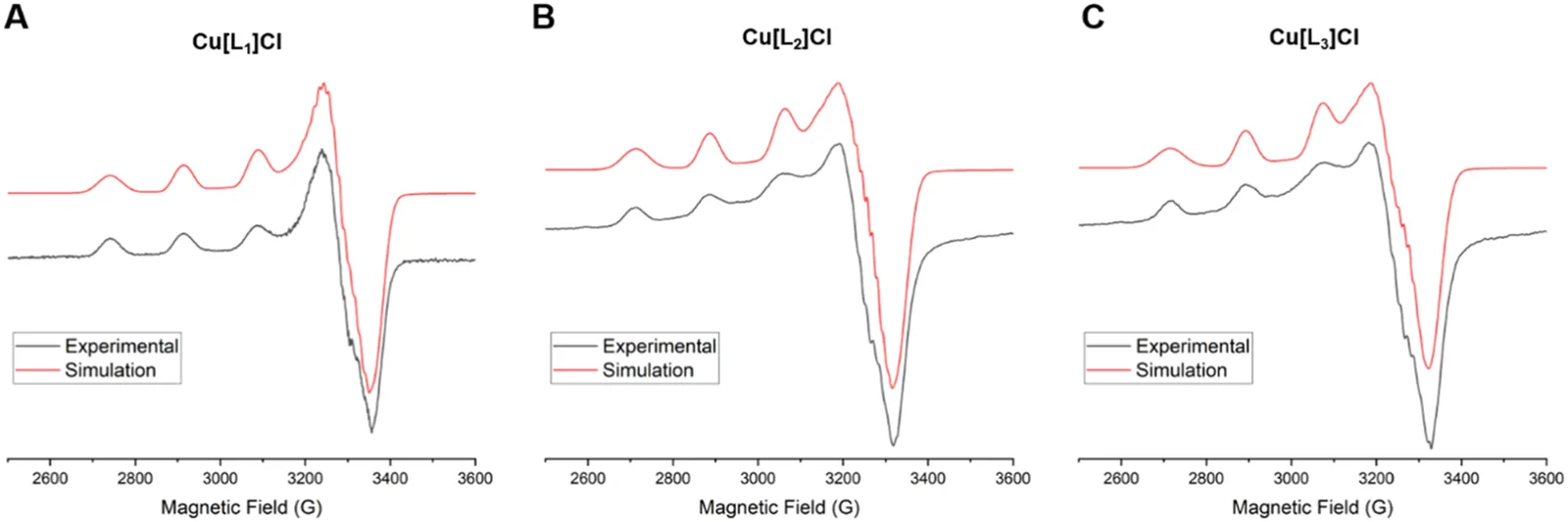
X-band EPR spectra (in black) and corresponding simulations (in red) of A) **Cu[L**_**1**_**]Cl**, B) **Cu[L**_**2**_**]Cl** and C) **Cu[L**_**3**_**]Cl**. The spectra are plotted as normalised intensities.

### Cell assays

To assess potential effects on cellular viability conferred by the complexes **Cu[L**_**1–3**_**]Cl**, cell lines derived from the GBM invasive margin (known as GIN cells) were exposed to each of the three complexes. These cells model the tumour population that remains after surgical removal, making them a clinically-relevant platform for testing potential therapeutics. Control experiments using just the ligands or copper alone were carried out to compare the activity of the complexes to their components.

As displayed in Fig. 5A–D, all three copper complexes exhibited impaired metabolic activity across the four GIN cell lines tested, with comparable IC_50_ values observed for each compound (Fig. 5E; all values between 2.5–10 μM). A one-way ANOVA followed by Tukey's multiple comparison test confirmed that there were no significant differences in efficacy among the complexes across any of the cell lines (*p* > 0.05). These values align well with other known cytotoxic small molecule drugs, such as doxorubicin, which exhibited IC_50_ values of 2.67–7.49 μM in 2D culture^33^).

**Fig. 5:**
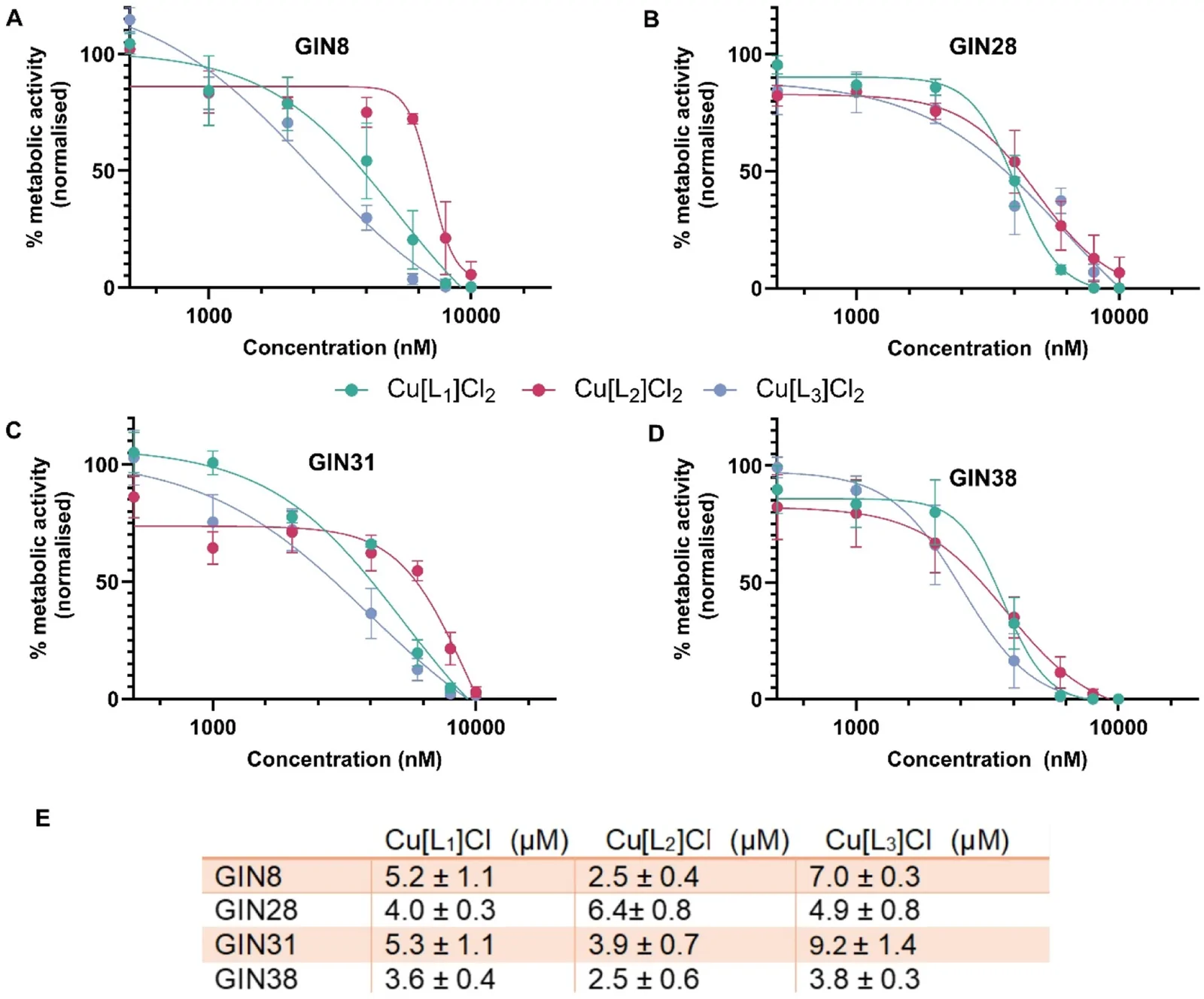
IC_50_ curves for **Cu[L**_**1**_**]Cl**, **Cu[L**_**2**_**]Cl** and **Cu[L**_**3**_**]Cl** against A) GIN8, B) GIN28, C) GIN31 and D) GIN38 cell lines. E) Corresponding IC_50_ values for each compound in all four cell lines. All data shown as mean ± SEM, *n* = 3.

Moreover, neither the free ligands nor copper alone conferred impaired metabolic activity, indicating that the observed anti-cancer phenotype is specifically driven by the formation of the copper–ligand complexes (Fig. S2A–D). This also provides assurance that neither the redox-active copper, nor the individual ligands, interfere with the assay. Copper complexes display enhanced biological activity compared to unbound copper due to their strong coordination with ligands, which stabilises their geometries.^34^ In addition, ligand coordination appears to increase their lipophilicity, thereby improving their ability to cross cellular membranes.^10^

The copper complexes were also evaluated in a primary human astrocyte line as a healthy-brain control (Fig. S3). The compounds reduced astrocyte metabolic activity to a degree comparable to their effects in GIN cells. Although a differential toxicity profile would be desirable to establish a therapeutic window, this astrocyte model does not recapitulate the phenotype of mature adult astrocytes present in glioblastoma patients. This limitation is important because foetal-derived or developmentally immature astrocyte lines typically display higher proliferative capacity, altered redox handling, and distinct metabolic programmes compared with adult astrocytes.^35,36^ As a result, their sensitivity to copper-based agents may overestimate off-target toxicity. Incorporating more physiologically relevant controls, such as adult astrocytes, iPSC-derived astrocytes, or *ex vivo* non-tumour brain tissue, will be essential for determining whether these complexes truly possess a therapeutic window in the clinical setting.

2D cell culture models do not fully capture *in vivo* complexity due to their growth as flat, rigid monolayers, often leading to overestimation of a therapeutic's activity.^37^ Therefore, the copper complexes were also evaluated in 3D spheroid cultures to assess their activity in a more physiologically relevant system and better mimic drug-tissue penetration. 3D culture allows intercellular interaction, cellular growth and spatial organisation in three dimensions.^38^ These experiments demonstrated that the copper complexes retain their cytotoxic effects in representative 3D spheroids, indicating that their activity extends beyond traditional 2D culture conditions (Fig. 6A–C). IC_50_ values remained in the low μM range, as shown in Table 2, whereby only a slight increase is demonstrated when comparing 2D to 3D IC_50_ values. Similarly to the results of the 2D culture, no significant differences are observed between the three complexes, suggesting that the inclusion of electron withdrawing or donating groups *para* to the hydroxyl group of salicylaldehyde confers no anti-cancer benefit in this experimental context.

**Fig. 6:**
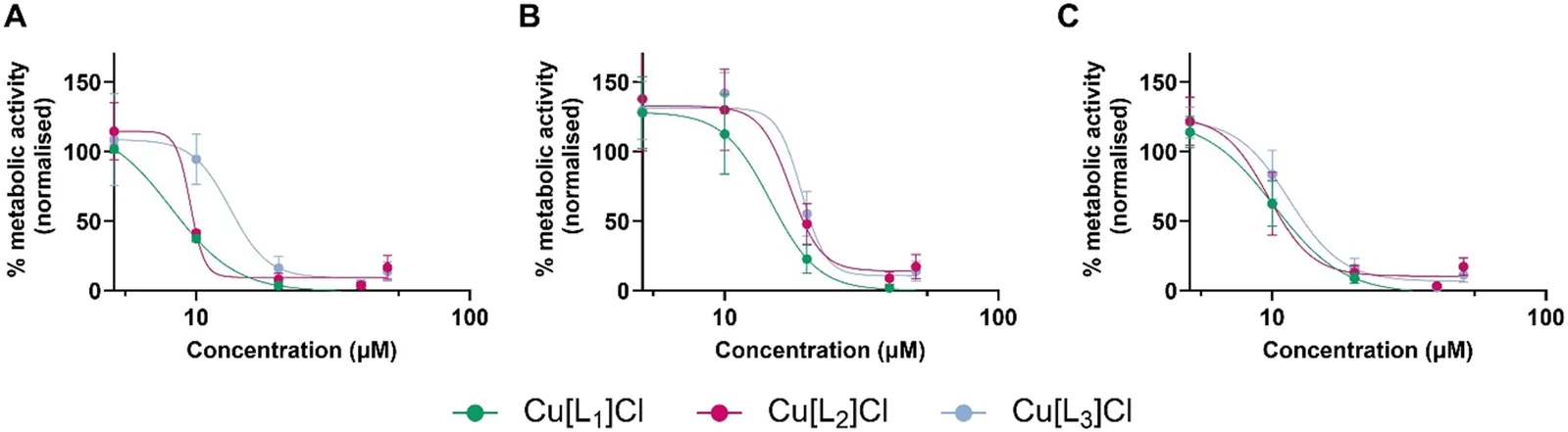
IC_50_ curves of GIN spheroids cultured with each of the **Cu[L**_**1–3**_**]Cl** complexes. Each complex was tested against A) GIN8, B) GIN28 and C) GIN31 cell lines. Results shown as mean ± SEM, *n* = 3.

**Table 2:** IC_50_ values for the **Cu[L**_**1–3**_**]Cl** complexes when tested in 3D spheroids. Results displayed as mean ± SEM, *n* = 3

	IC_50_ (μM)		
	**Cu[L**_**1**_ **]Cl**	**Cu[L**_**2**_ **]Cl**	**Cu[L**_**3**_ **]Cl**
GIN8	8.1 ± 0.7	9.5 ± 0.9	13.3 ± 0.9
GIN28	14.7 ± 1.3	17.4 ± 1.0	19.1 ± 0.2
GIN31	10.2 ± 1.5	9.7 ± 1.2	11.6 ± 1.4

Once impaired cellular metabolic activity was confirmed, initial investigations on the potential mechanism of action of the copper complexes were carried out. Reactive oxygen species (ROS) are strongly associated with transition metal ions, with hydroxyl radicals and superoxide ions being the primary species generated.^39^ To assess ROS production, GIN31 cells were incubated with the cell-permeable probe: 5-(and-6)-chloromethyl-2′,7′-dichlorodihydrofluorescein diacetate, acetyl ester (CM-H_2_DCFDA), which is cleaved by intracellular esterases and subsequently oxidised by ROS to form the green fluorescent compound 2′,7′-dichlorofluorescein (DCF). The results, displayed in Fig. 7A–C, demonstrate a significant increase in cellular ROS in the presence of each copper complex. Images showing this fluorescence in cells in the presence of **Cu[L**_**1**_**]Cl** is shown in SI Fig. S4. However, no difference was noted between each complex, in line with the IC_50_ results, suggesting that changing the ligand does not impact the cellular ROS generation.

**Fig. 7:**
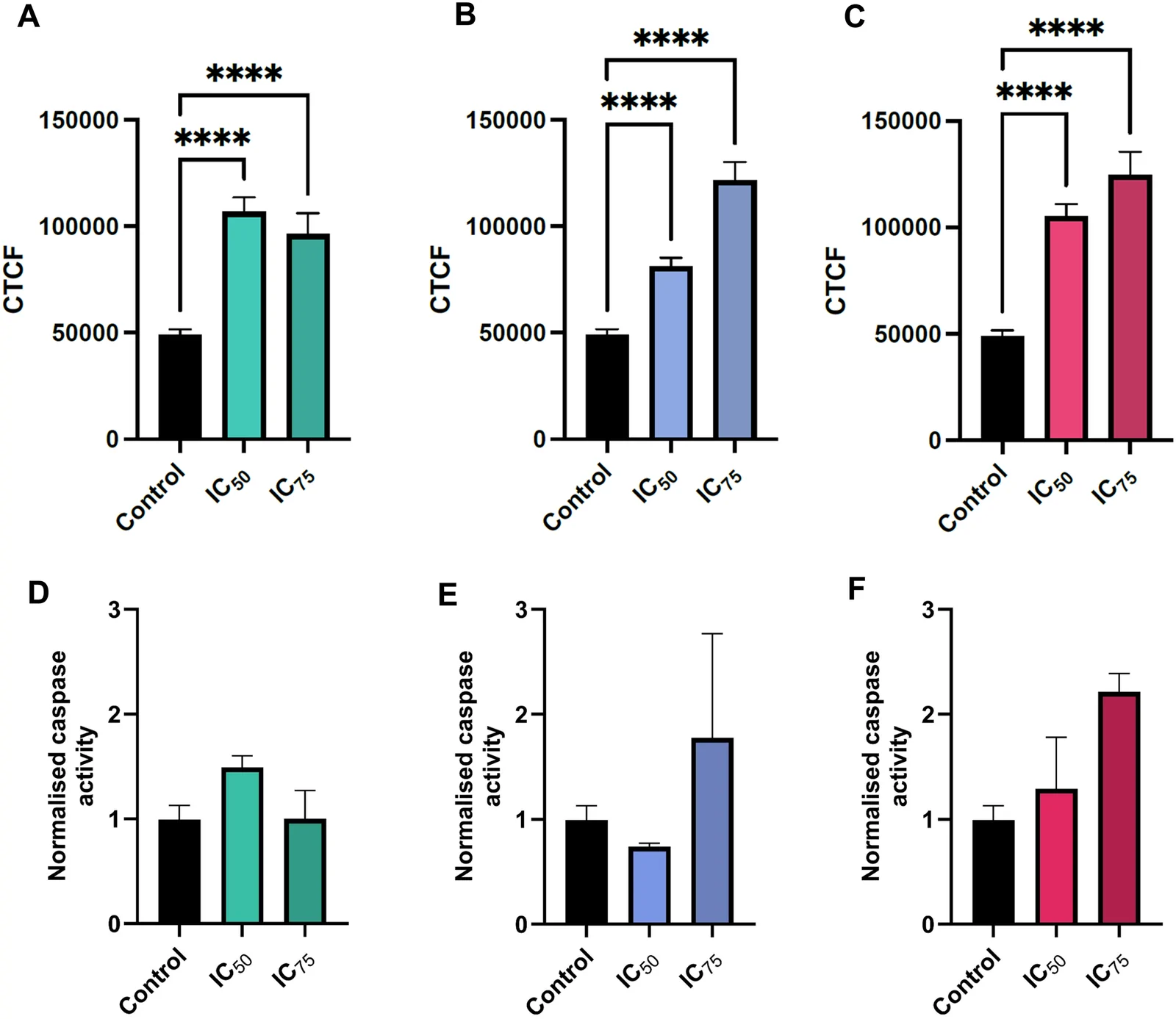
ROS assays showing a significant increase in ROS 4 hours after treatment of IC_50_ and IC_75_ concentration of A) **Cu[L**_**1**_**]Cl**, B) **Cu[L**_**2**_**]Cl** and C) **Cu[L**_**3**_**]Cl**. Results displayed as mean ± SEM, *n* = 3. Caspase 3/7 assays showed no significant increase in cleaved caspase 3/7 in GIN31 cells in the presence of IC_50_ and IC_75_ concentrations of D) **Cu[L**_**1**_**]Cl**, E) **Cu[L**_**2**_**]Cl** and F) **Cu[L**_**3**_**]Cl**, indicating that the copper complexes do not significantly trigger the apoptotic pathway. Results displayed as mean ± SEM, *n* = 2.

Caspase 3/7 activity was then assessed in GIN31 cells to identify whether apoptotic cell death is induced by the copper complexes. As caspases 3 and 7 function as key executioner enzymes in the apoptotic pathway,^40^ their activation serves as an indicator of apoptosis. Fig. 7D–F displays caspase 3/7 activity (normalised to untreated control cells) in response to **Cu[L**_**1–3**_**]Cl** at 6 hours following treatment, which showed a marginal elevation at some concentrations, although statistical significance was not achieved. The 6-hour time point was selected as the optimal assessment window, as little response to a positive control (doxorubicin) was observed at either 3 or 24 hours, suggesting that these time points preceded and exceeded the peak response, respectively. In contrast, a clear response was observed at 6 hours. Analysis of two biological replicates, each performed with four technical replicates, revealed no significant cleaved caspase-3/7 activity in response to any of the complexes. However, the data does not exclude an alternative mode of cell death conferred by these copper complexes. Other ROS-induced cell death mechanisms exist beyond apoptosis. For example, ferroptosis has been linked with ROS activity due to the disruption of intercellular iron homeostasis and lipid peroxide metabolism.^41^ Type II programmed cell death, autophagy, has also been linked with increased ROS levels *via* copper complexes.^42^ Pyroptosis, a form of programmed inflammatory cell death, is caused by the activation of inflammasomes. ROS are upstream activators of the NLRP3 inflammasome, thus promoting this process. Furthermore, oxeiptosis is a caspase-independent non-inflammatory cell death mechanism whereby ROS activates the Keap1-phosphoglycerate mutase family member 5-apoptosis inducing factor mitochondria associated 1 (PGAM5-AIFM1) axis.^43^ Consequently, this suggests there are many mechanisms by which these *N*,*N*,*O*-tridentate Schiff base copper(ii) complexes could lead to invasive GBM cell death.

## Conclusion

Three novel Schiff base ligands and their corresponding copper(ii) complexes were synthesised, characterised and tested for their potential use against GBM. The ligands include a salicylaldehyde moiety with non-substituted, electron-withdrawing or electron-donating substituent *para* to the metal-coordinating hydroxyl group to evaluate the effect of these groups on the activity. All three complexes are readily formed upon addition of Cu(ii) to the tridentate ligands, with the metal ion adopting a square planar geometry in all cases, as confirmed by EPR spectroscopy. The complexes induce marked metabolic impairment in GBM invasive margin cells across both 2D and 3D culture systems, with IC_50_ values in the range of 2.5–10 μM and 8–20 μM for 2D and 3D cultures, respectively. These results are in line or better than other reported Cu complexes tested against glioblastoma cell lines.^23^ These complexes trigger a marked increase in ROS production, and caspase-3/7 analysis indicates that metabolic impairment is not significantly associated with at least one mode of apoptosis. Variations in ligand structure did not produce notable differences in cellular responses, suggesting that the individual ligands do not impact the mode of action of the complexes. Further studies are required to clarify the precise mechanism of action of these complexes in GBM cells. Although metabolic impairment and ROS-inducing capacity have been established, the downstream pathways associated with this phenotype remain to be defined. Notwithstanding, these complexes show high potency against these hard-to-treat invasive cancer cells, providing promise as a potential therapeutic against this dismal disease. As a result, comparing the activity of these complexes against other cancer types could reveal whether they have broad cytotoxic activity.

## Ethics statement

Experiments using primary cell lines derived from human brain tumour tissue were approved by the National Health Service Research and Ethics Committee East Midlands (Ref: 11/EM/0076), and such research is subject to audits by the Human Tissue Authority. Informed consents were obtained from human participants. All samples were handled and monitored in accordance with national guidelines of the UK Human Tissue Act 2004, specifically ‘Part 2 – Regulation of activities involving human tissue’.

## Author contributions

Conceptualisation: LC, PM. Methodology and investigation: TW, KM. Data curation and formal analysis: TW, KM, SA, SM, DS, KPK, CM, UK and HP. Writing – original draft: TW, RR, LC and PM. Writing – review & editing: LC, PM.

## Conflicts of interest

There are no conflicts to declare.

## Supplementary Material

MD-OLF-D6MD00801A-s001

MD-OLF-D6MD00801A-s002

## Data Availability

Analytical and characterisation data for **L**_**1–3**_ and **Cu[L**_**1-3**_**]Cl** can be accessed through the University of Nottingham depository, DOI: https://doi.org/10.17639/nott.34864. Supplementary information (SI): raw data is in the .XLSX file and SI figures and tables are in the .PDF file. See DOI: https://doi.org/10.1039/d6md00801a. CCDC 2421573–2421576 and 2571886 contain the supplementary crystallographic data for this paper.^44*a–e*^
