# Supplementary material for: Copper complexes as candidate therapeutic agents against infiltrative glioblastoma cells
Source: RSC Med Chem. 2026 Oct 15. Online ahead of print. doi: 10.1039/d6md00801a (PMC13702835; doi:10.1039/d6md00801a)
Supplement: Supplementary file 1 [file MD-OLF-D6MD00801A-s002.pdf]

Supplementary Information (SI) for RSC Medicinal Chemistry.
This journal is © The Royal Society of Chemistry

Supplementary Information for

Copper complexes as candidate therapeutic agents against infiltrative glioblastoma cells

Thomas Weston[1], Kayley Mulhall[2], Sophie Melling[2], Dylan Smith[2], Stephen P. Argent[1], Konstantinos-Panagiotis Karadimas[2], Cara Moloney[3], Une Kontrimaite[2], Harry Porter[2], Ruman Rahman[2], Luisa Ciano[1]* and Phoebe McCrorie[2]*

[1] School of Chemistry, University Park, University of Nottingham, NG7 2RD, UK

[2] Biodiscovery Institute, School of Medicine, University Park, University of Nottingham, NG7 2RD, UK

[3] School of Pharmacy, University Park, University of Nottingham, NG7 2RD, UK

* Corresponding author: luisa.ciano@nottingham.ac.uk

Phoebe.McCrorie1@nottingham.ac.uk

Supplementary Tables

Table S1. Combined CIF reports for ligands L_2 and L_3 and coordination compounds Cu[L_1]Cl and Cu[L_3]Cl.

	**L_2-H_2O**	**L_2**	**L_3**	**Cu[L_1]Cl**	**Cu[L_3]Cl**
Chemical formula	$(C_{13}H_{10}Cl_2N_4O)_2 \cdot H_2O$	$C_{13}H_{10}Cl_2N_4O$	$C_{14}H_{13}ClN_4O_2$	$C_{13}H_{10}Cl_2CuN_4O \cdot CH_4O$	$C_{14}H_{12}Cl_2CuN_4O_2 \cdot H_2O$
M_r	317.65	309.15	304.73	404.73	420.73
Crystal system	Monoclinic	Monoclinic	Orthorhombic	Monoclinic	Orthorhombic
Space group	*C*2/*c*	*P*2₁/*c*	*P*2₁2₁2₁	*P*2₁/*c*	*Pbca*
a, *b*, *c* (Å)	30.9433 (6), 7.1495 (2), 12.1772 (2)	5.8777 (1), 19.9383 (3), 11.2632 (1)	5.0471 (1), 11.7552 (3), 23.1569 (6)	11.7162 (6), 15.3768 (8), 17.7976 (11)	15.8038 (1), 13.2883 (1), 30.9004 (2)
α, β, γ (°)	90, 93.864 (2), 90	90, 95.338 (1), 90	90, 90, 90	90, 97.510 (6), 90	90, 90, 90
V (Å^3)	2687.83 (10)	1314.23 (3)	1373.89 (6)	3178.9 (3)	6489.26 (8)
Z	4	4	4	8	16
Radiation	Cu *K*α	Cu *K*α	Cu *K*□	Cu *K*α	Cu *K*α
m (mm^{-1})	4.41	4.46	2.56	5.16	5.13
Temperature / K	120	120	120	120	120
Crystal size (mm)	0.37 × 0.13 × 0.08	0.36 × 0.14 × 0.06	0.28 × 0.05 × 0.01	0.21 × 0.03 × 0.01	0.14 × 0.02 × 0.02
Diffractometer	SuperNova, Titan S2	SuperNova, Atlas S2	XtalLAB PRO *MM007*, PILATUS3 R 200K	SuperNova, Titan S2	XtalLAB PRO *MM007*, PILATUS3 R 200K
T_{min}, T_{max}	0.327, 1.000	0.410, 1.000	0.648, 1.000	0.616, 1.000	0.751, 1.000

No. of measured, independent and observed [I > 2s(I)] reflections	20443, 2649, 2493	15689, 2558, 2406	4949, 2521, 2338	13659, 6120, 4397	137451, 6741, 6293
R_{int}	0.025	0.027	0.033	0.079	0.038
(sin q/l)$_{max}$ (Å^{-1})	0.619	0.619	0.629	0.619	0.629
R[F^2 > 2s(F^2)], wR(F^2), S	0.025, 0.067, 1.08	0.027, 0.075, 1.05	0.047, 0.138, 1.08	0.067, 0.174, 1.01	0.029, 0.077, 1.08
No. of reflections	2649	2558	2521	6120	6741
No. of parameters	198	190	200	435	459
No. of restraints	4	2	3	6	6
Dr$_{max}$, Dr$_{min}$ (e Å^{-3})	0.29, -0.29	0.27, -0.28	0.88, -0.40	1.06, -0.76	0.47, -0.47
Absolute structure parameter (Flack)	–	–	-0.002 (15)	–	–
CCDC	2421573	2571886	2421574	2421575	2421576

Table S2. Simulated X-band EPR parameters for copper(II) complexes.

		Cu[L_1]Cl	Cu[L_2]Cl	Cu[L_3]Cl
g values	g_1	2.044	2.043	2.047
	g_2	2.070	2.08	2.081
	g_3	2.248	2.242	2.236
A_{Cu} (MHz)	$\|A_1\|$	15	40	25
	$\|A_2\|$	50	70	70
	$\|A_3\|$	534	535	546
A_N (isotropic) (MHz)		35, 27	35, 35	35, 35
A_{Cl} (isotropic) (MHz)		40	38	38
A_{Cu} strains (MHz)		30, 40, 80	40, 80, 100	50, 80, 120
Line widths (mT)		0.4, 0.4	0.5, 0.5	0.4, 0.4
Frequency (GHz)		9.439735	9.329548	9.330473

Supplementary Figures

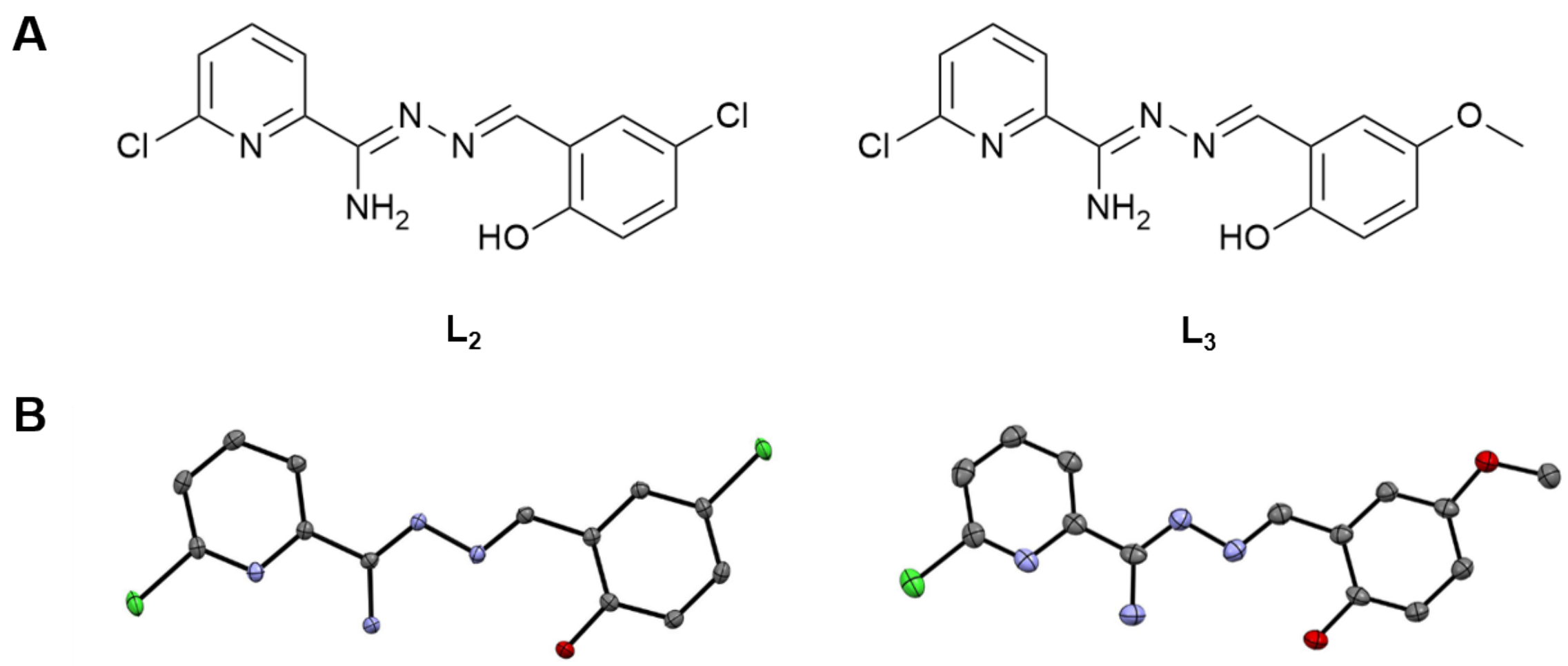

Figure S1. A) Structure of L_2 and L_3. **B)** Ortep diagrams of L_2 (left) and L_3 (right). Hydrogen atoms have been omitted for clarity. Carbon atoms are grey, nitrogen blue, oxygen red and chloride green.

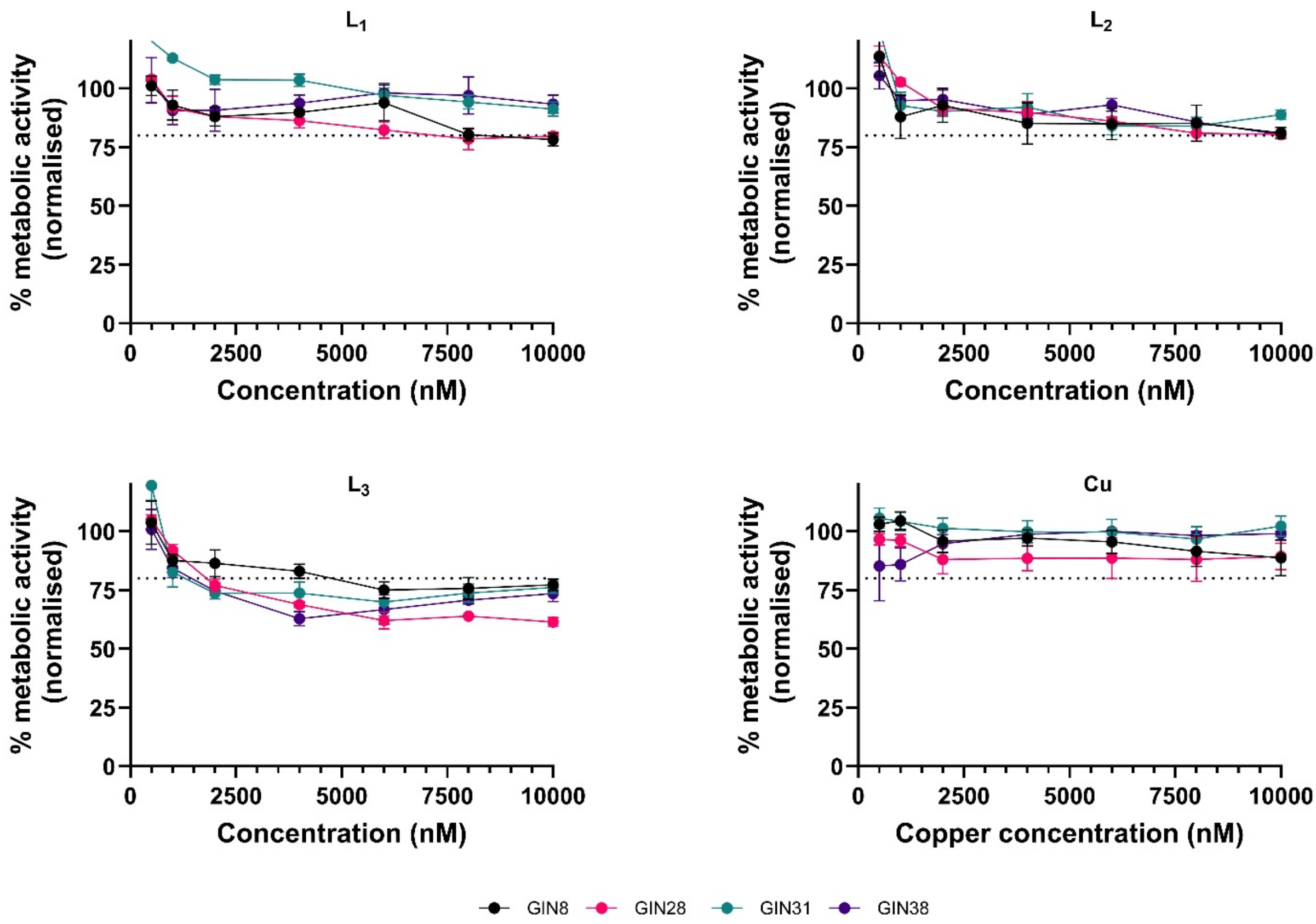

Figure S2. Analysis of metabolic activity in GIN8, GIN28, GIN31 and GIN38 in the presence of L_1 (A), L_2 (B), L_3 (**C**) and copper only (D). Dotted line set at 80% to allow for visualisation. All data shown as mean ± SEM, *n*=3

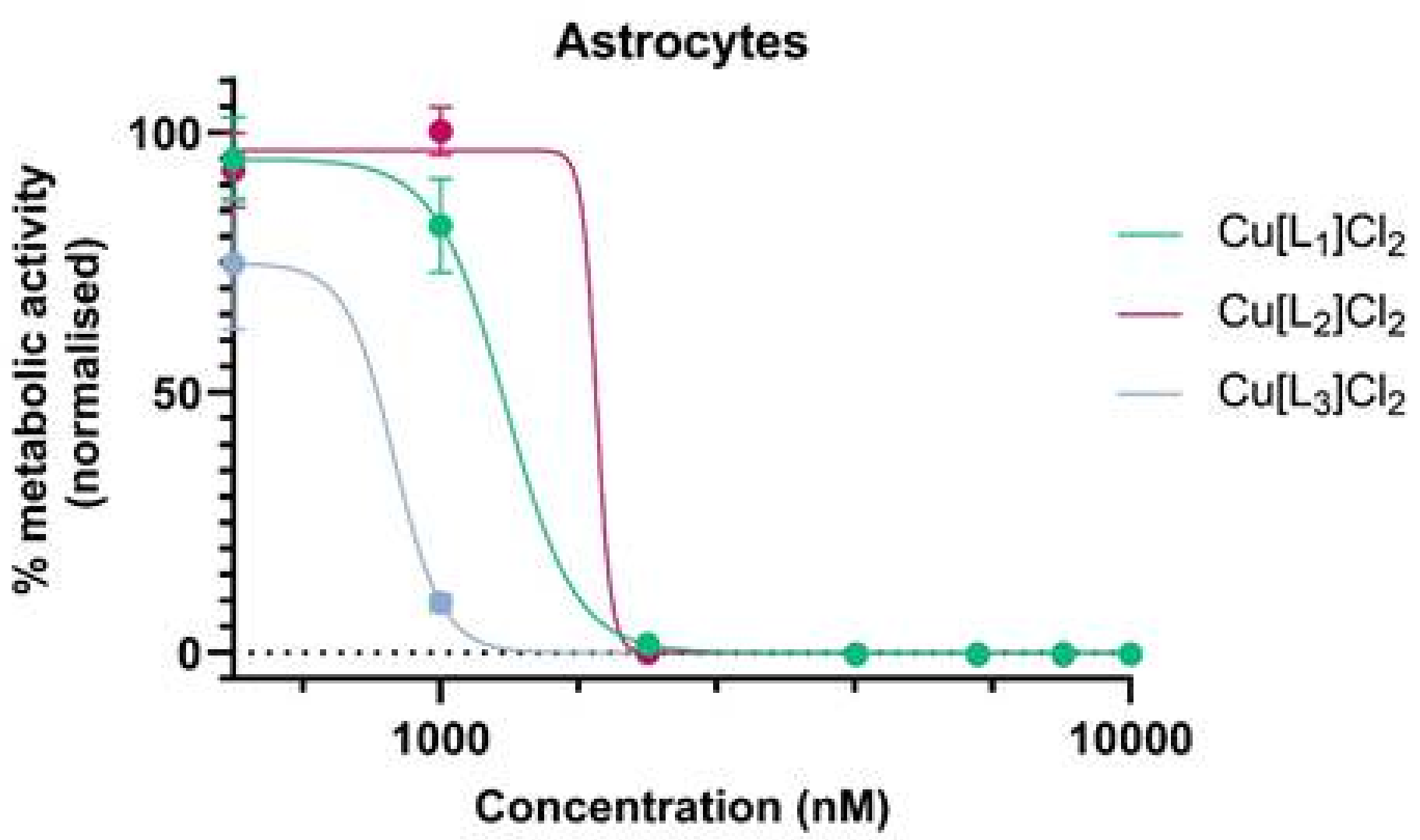

Figure S3. Assessment of copper complexes on human astrocytes. Data shown as mean ± SEM.

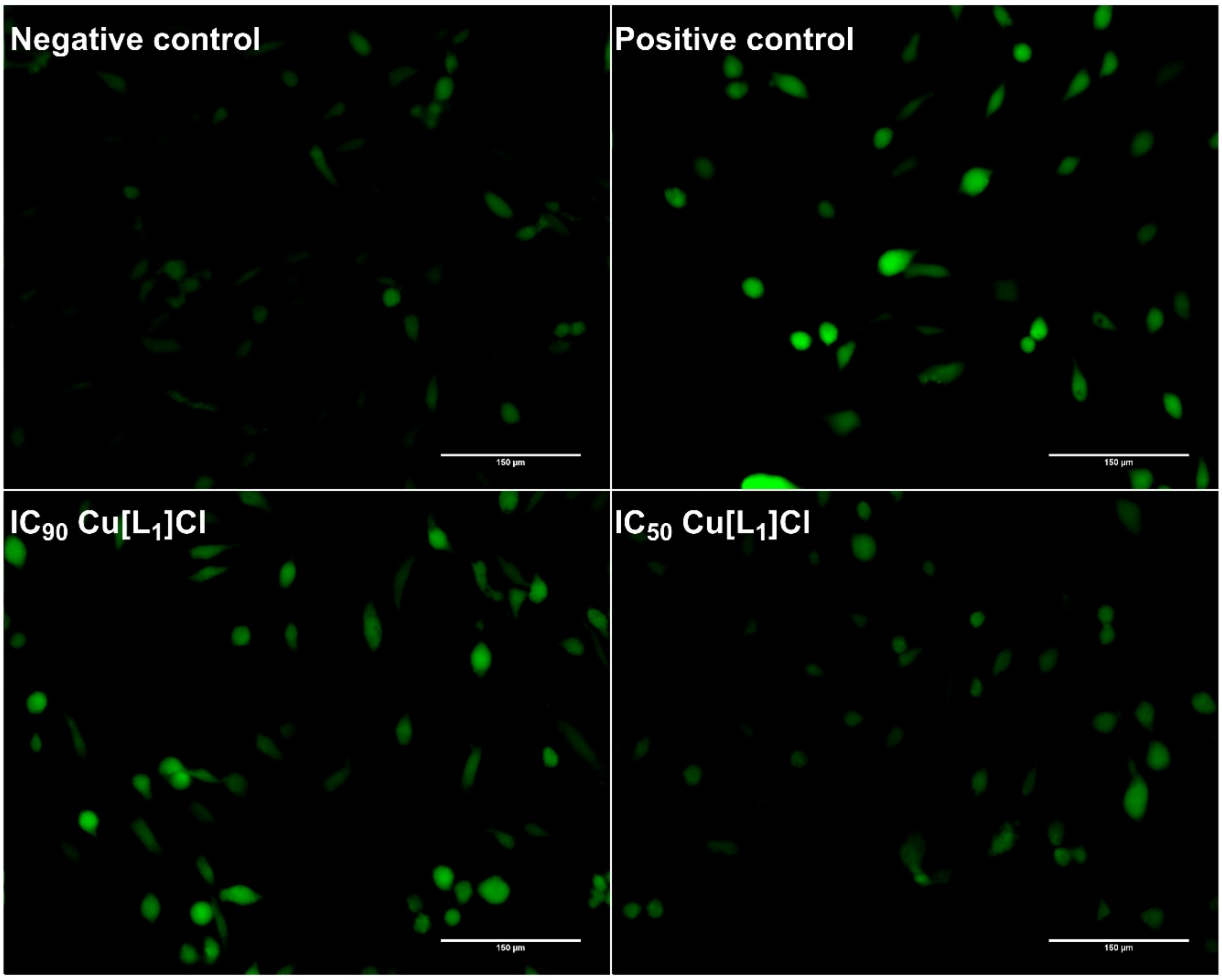

Figure S4. Images demonstrating reactive oxygen species activity in GIN31 cells in the presence of **Cu[L$_1$]Cl** at IC_{50} and IC_{90}. H_2O_2 was used as a positive control. Error bars = 150 μm.
